# Supplementary material for: The distribution of fitness effects during adaptive walks using a simple genetic network
Source: PLoS Genet. 2024 May 24;20(5):e1011289. doi: 10.1371/journal.pgen.1011289 (PMC11156440; doi:10.1371/journal.pgen.1011289)
Supplement: S1 Table — Table of symbols, names, descriptions, and values for relevant parameters used in the NAR model. (PDF) [file pgen.1011289.s002.pdf]

S1 Table. NAR model parameters. Table of symbols, names, descriptions, and values for relevant parameters used in the NAR model.

Symbol	Parameter	Description
**Molecular components**		
α_Z	Z removal rate	The rate at which Z product is removed from the cell.
β_Z	Z production rate	The rate at which Z is produced.
K_Z	Repression coefficient	The Z product concentration at which further Z expression is reduced by half. Fixed at $K_Z = 1$.
K_{XZ}	Activation coefficient	The X product concentration at which Z expression is half the maximum. Fixed at $K_{XZ} = 1$.
h	Hill coefficient	The steepness coefficient of the Z activation/repression curve. Fixed at $h = 8$.
**Integration parameters**		
t_{start}	X activation time	The left boundary where $X = 1$ when solving the NAR ODE. Before this point, $X = 0$.
t_{stop}	X deactivation time	The right boundary where $X = 1$ when solving the NAR ODE. After this point, $X = 0$.
t_{max}	Development time	The amount of time for which $Z(t)$ is solved for. Models the developmental time of the trait in an individual.
**Model abbreviations/definitions**		
$mQTL$	Molecular quantitative trait locus	A QTL for a molecular component.
$\mathbf{C}$	Molecular components	A vector of ODE coefficients contributing to Z expression. Includes α_Z and β_Z.
w	Relative fitness	Relative fitness of an individual.
$\mathbf{P_O}$	Phenotypic optimum	The phenotype where $w = 1$.
σ	Fitness function width	The width of the Gaussian fitness function. A measure of selection strength.
a	Allelic effect	The allelic effect of an mQTL on a molecular component. Note this is different from s, which is the effect of an allele on fitness. It also is not a measure of the allelic effect on the phenotype due to the ODE intermediate between a and the phenotype.
s	Selection coefficient	The effect of a mutation on fitness.
