# Supplementary material for: The distribution of fitness effects during adaptive walks using a simple genetic network
Source: PLoS Genet. 2024 May 24;20(5):e1011289. doi: 10.1371/journal.pgen.1011289 (PMC11156440; doi:10.1371/journal.pgen.1011289)
Supplement: S2 Table — Mean generalized Pareto distribution (GPD) parameters fit to mutant screen distributions of fitness effects over adaptive walks in additive and network populations. Brackets indicate 95% confidence intervals. Parameters were estimated by fitting a GPD to a random sample of mutations from the mutant screen experiments conducted on each replicate at each adaptive step. κ is the shape parameter of the GPD. κ¯ is the mean κ value across n replicate simulations. The log-likelihood ratio tests the null hypothesis that a sample of beneficial mutations belongs to an exponential distribution (which meets κ = 0). P-values across the n replicates were combined using Fisher’s method. (PDF) [file pgen.1011289.s003.pdf]

S2 Table. Generalized Pareto distribution parameter estimates. Mean generalized Pareto distribution (GPD) parameters fit to mutant screen distributions of fitness effects over adaptive walks in additive and network populations. Brackets indicate 95% confidence intervals. Parameters were estimated by fitting a GPD to a random sample of mutations from the mutant screen experiments conducted on each replicate at each adaptive step. κ is the shape parameter of the GPD. $\bar{\kappa}$ is the mean κ value across n replicate simulations. The log-likelihood ratio tests the null hypothesis that a sample of beneficial mutations belongs to an exponential distribution (which meets $\kappa = 0$). P-values across the n replicates were combined using Fisher's method.

**Additive model**				
**Adaptive Step**	n	$\bar{\kappa}$	**Log-likelihood ratio**	**p-value**
Before optimum shift	1213	$-2.12(\pm 0.04)$	$0.73(\pm 0.003)$	< 0.001
1	1014	$-2.12(\pm 0.04)$	$0.83(\pm 0.004)$	< 0.001
2	275	$-2.00(\pm 0.08)$	$0.86(\pm 0.007)$	< 0.001
≥ 3	39	$-1.92(\pm 0.15)$	$0.88(\pm 0.017)$	< 0.001
**Network model**				
**Adaptive Step**	n	$\bar{\kappa}$	**Log-likelihood ratio**	**p-value**
Before optimum shift	950	$-1.88(\pm 0.03)$	$0.77(\pm 0.002)$	< 0.001
1	833	$-2.03(\pm 0.04)$	$0.83(\pm 0.004)$	< 0.001
2	214	$-2.12(\pm 0.11)$	$0.87(\pm 0.006)$	< 0.001
≥ 3	38	$-2.11(\pm 0.18)$	$0.88(\pm 0.012)$	< 0.001
