# Supplementary figures and images for: The distribution of fitness effects during adaptive walks using a simple genetic network
Source: PLoS Genet. 2024 May 24;20(5):e1011289. doi: 10.1371/journal.pgen.1011289 (PMC11156440; doi:10.1371/journal.pgen.1011289)

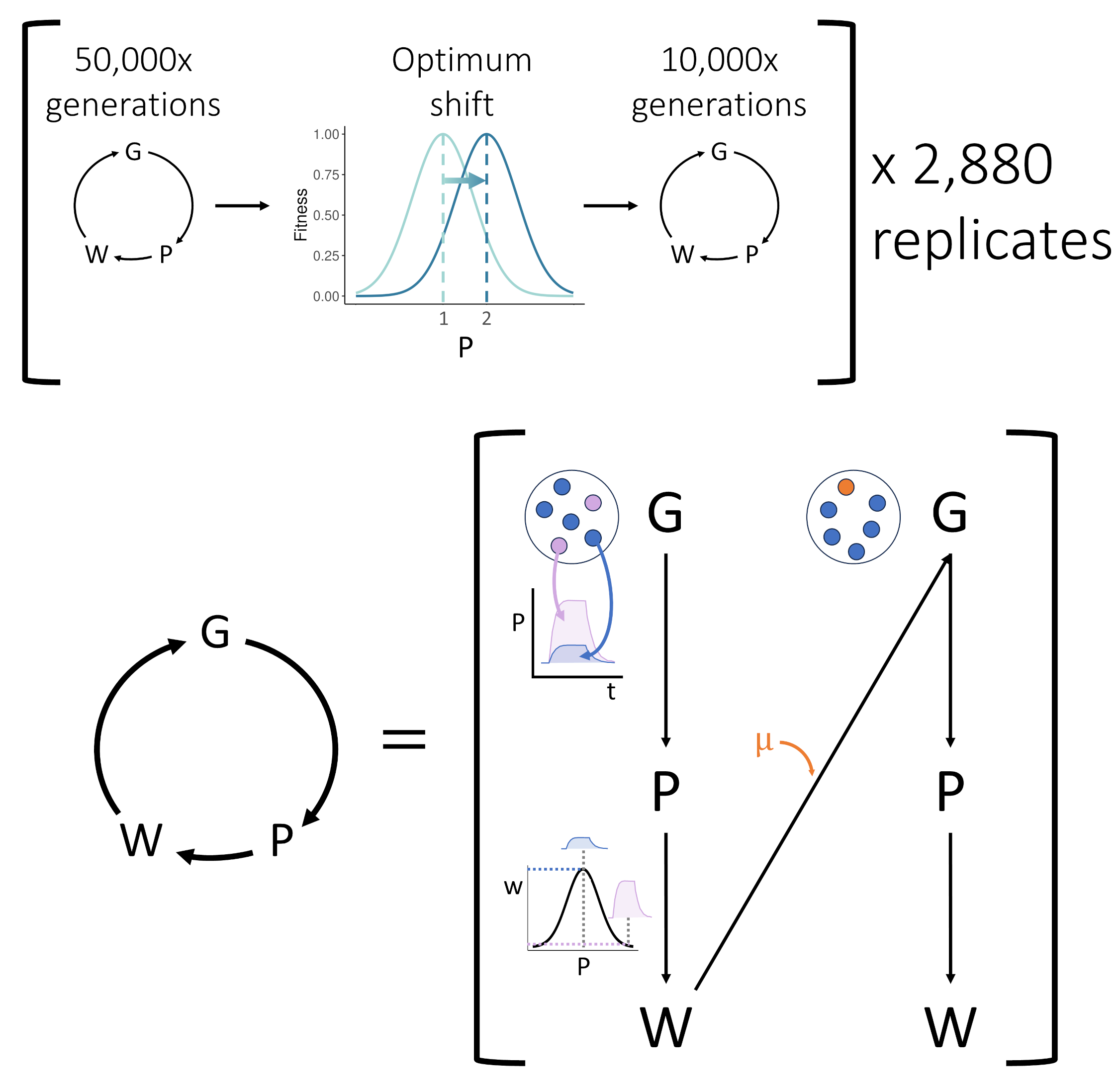

Supplement: S1 Fig — SLiM simulates a Wright-Fisher process to model evolutionary change through a genotype-phenotype-fitness (GPW) map. Our simulation began with 50,000 generations of burn-in to ensure populations were adapted to the environment. We then shifted the phenotypic optimum and adaptation was tracked for a further 10,000 generations. This process was repeated 2,880 times per model for replication purposes (i.e. there were 2,880 replicates of network and additive adaptive walks for a total 5,760 simulations). The GPW map consisted of several stages: first, the genotype was translated to phenotype via summing genetic effects at QTLs (for additive models) or by solving a system of ordinary differential equations (network models, P/t figure shows solutions to the differential equation for the blue and purple genotypes). Phenotype was then translated to fitness by a stabilizing selection fitness function (shown by the w/P figure—the purple phenotype has lower fitness than the blue phenotype). The fitness value then influenced the chance that an individual was sampled as a parent for the next generation. After parents were chosen, random mutations (μ) could occur to introduce further genotypic and phenotypic variation in the next generation (shown in orange). (TIFF) [file pgen.1011289.s004.tiff]

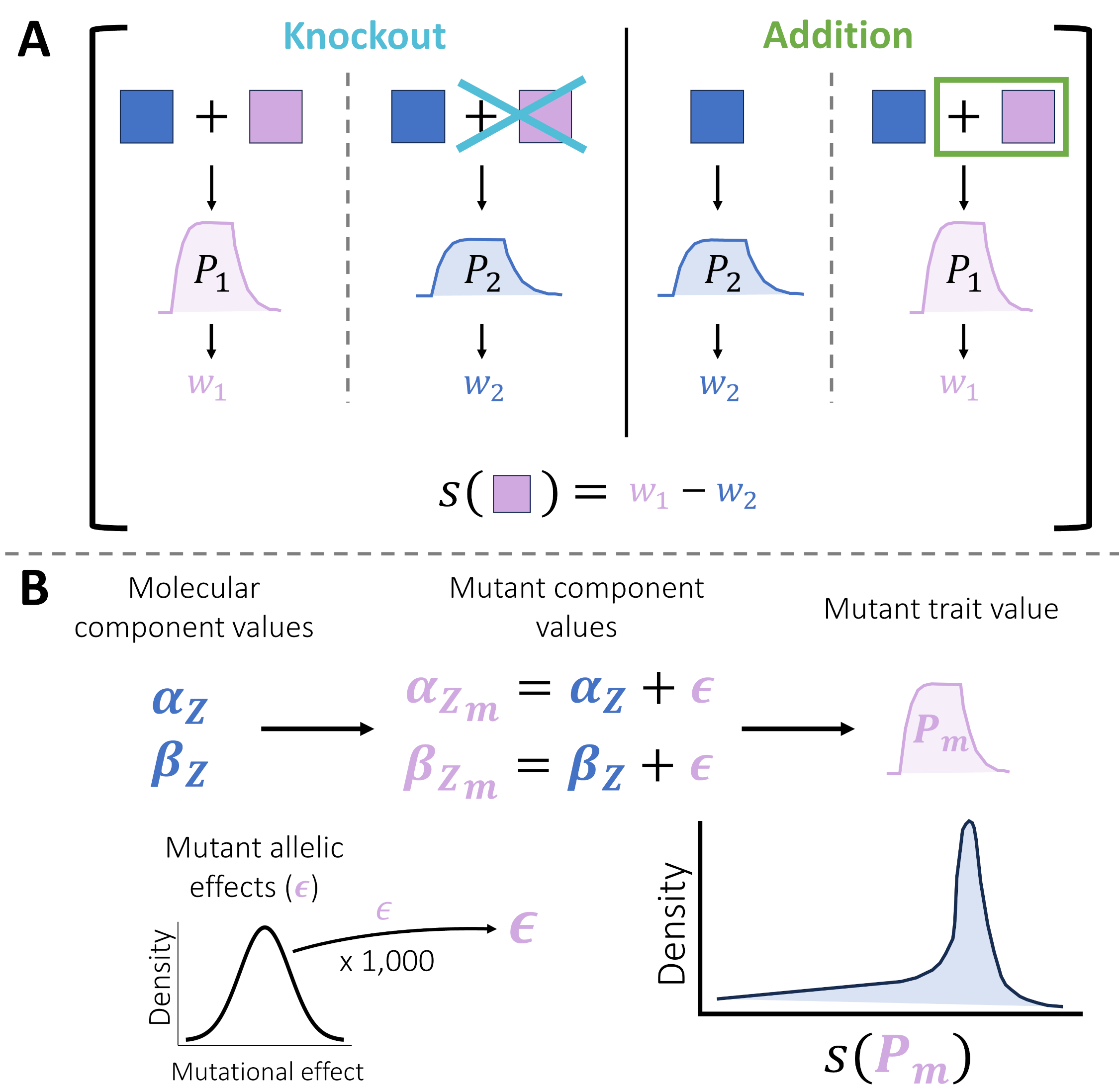

Supplement: S2 Fig — (A) Consider two alleles contributing to the phenotype (blue and purple boxes) at either the same or different loci. Individuals with both alleles have some phenotype (P1), which gives rise to a fitness (w1). By removing the purple allele and recalculating the phenotype, we achieve a different phenotype (P2) and fitness (w2). The difference between w1 and w2 represents the selection coefficient (s) of the purple allele. This difference can be measured either through addition—adding the purple allele to the genotype, or by knockout (removing the purple allele from the genotype). (B) To estimate the distribution of fitness effects (DFE) among new mutations, we conducted a mutation screen experiment. We generated mutants by taking 1,000 samples (ϵ) from a standard normal distribution and adding those to the molecular component values from the SLiM simulations (αZ and βZ). Mutant phenotypes (Pm) were calculated by inputting the mutant component values into an ordinary differential equation and solving it. The 1,000 samples were independently added to each molecular component to measure the DFE of both components. We then calculated the selection coefficients of ϵ by the addition method in (A). Pm represents P1 in (A), whilst the phenotypes without the added ϵ represents P2. We then plotted the joint distribution of s across all sampled ϵ. (TIFF) [file pgen.1011289.s005.tiff]

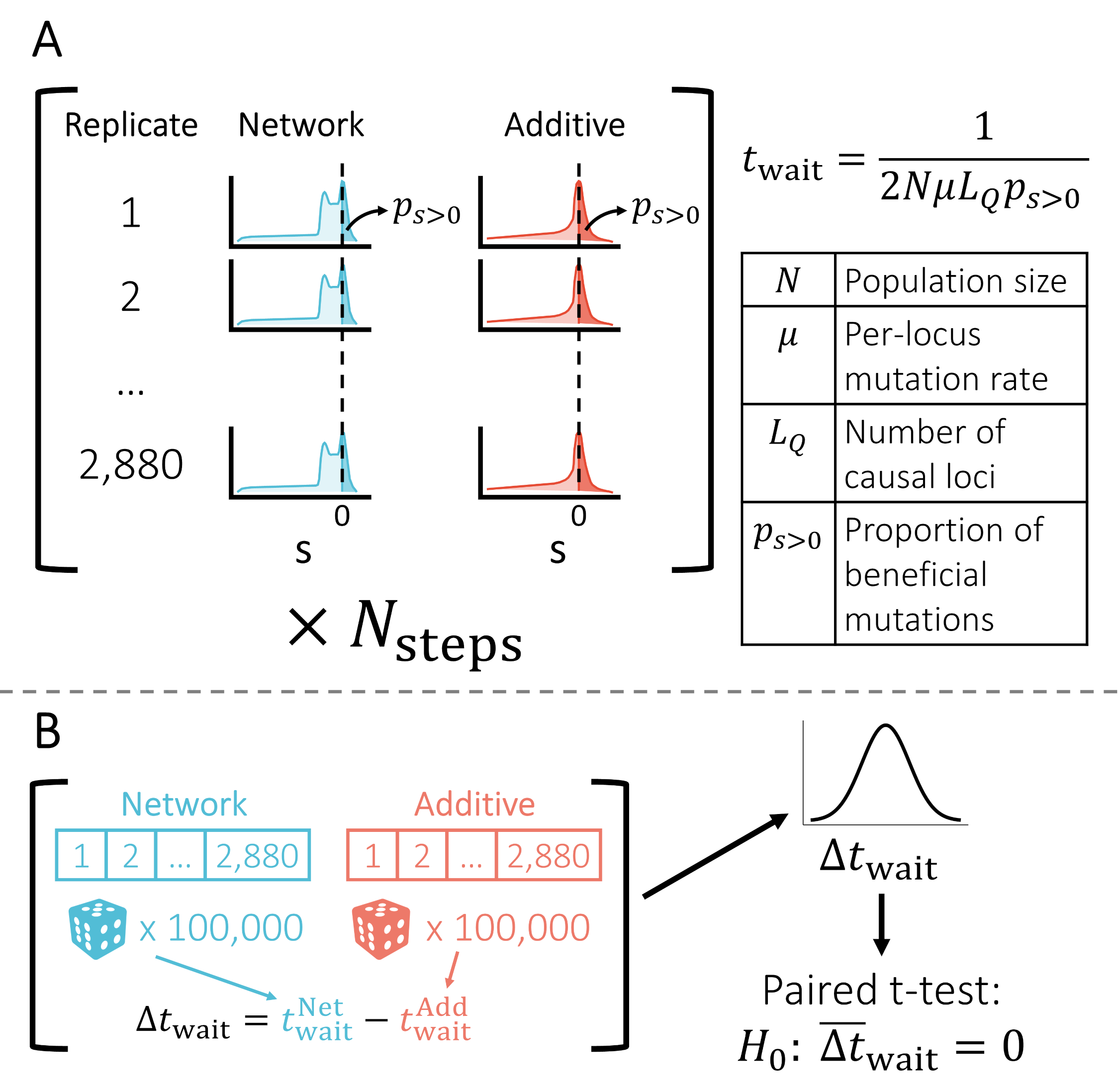

Supplement: S3 Fig — (A) For each of the 2,880 replicates, we took the distribution of s calculated during the mutation screen experiments and extracted the proportion of new mutations with s > 0, ps>0. This is the area under the curve shown in dark blue/red (to the right of the dashed line). This was repeated for each adaptive step (Nsteps within a replicate (i.e. replicates with more than one adaptive step had ps>0 calculated for each adaptive step). The waiting time to a new beneficial mutation for a given replicate at a given step was calculated as twait = 1/(2NμLQps>0), where N is the population size (multiplied by 2 because we simulate a diploid organism), μ is the per-locus, per-generation mutation rate, and LQ is the number of causal loci. (B) To calculate the difference between models in the waiting time to a new beneficial mutation, we ran a bootstrap analysis. We sampled 100,000 random pairs of additive and network models calculating twait for each. We calculated the difference between their waiting times, Δtwait. The random sampling gave a distribution of differences between network and additive waiting times. We used a paired t-test to determine if the mean difference in waiting times between models, Δt¯wait was not zero (and hence there was a difference between models). (TIFF) [file pgen.1011289.s006.tiff]

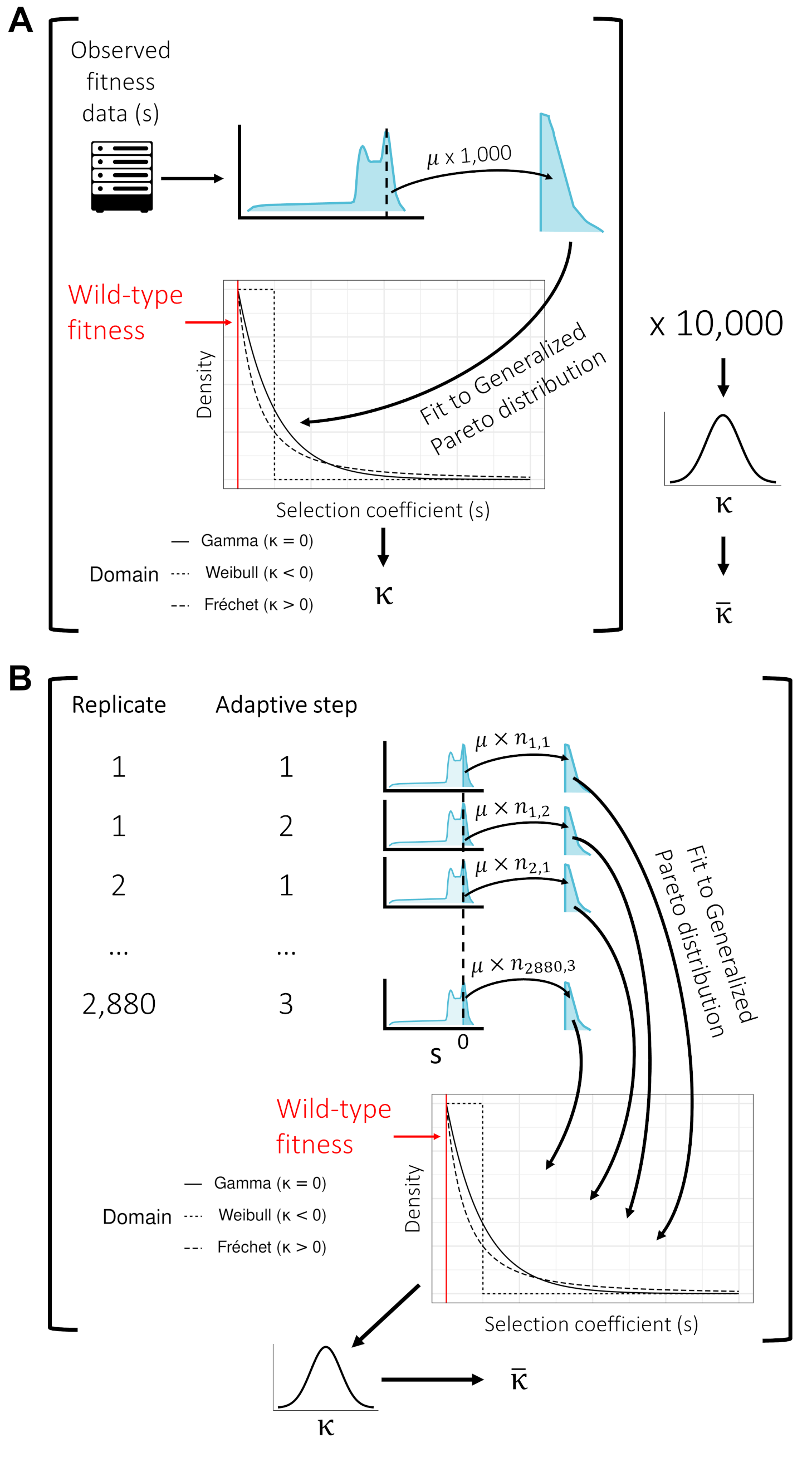

Supplement: S4 Fig — (A) Using the pooled distribution of s across all simulations and adaptive steps that we calculated during the mutation screen experiments, we sampled 1,000 beneficial mutations (where s > 0) to generate a distribution of beneficial mutations. We then fit this to a generalized Pareto distribution (GPD) using the method outlined by Beisel et al. [88]. From the fit, we extracted the shape parameter of the fit, κ. This process was repeated 10,000 times to generate a distribution of κ. We treated the mean κ, κ¯ as the estimate for the shape parameter. (B) We sampled nx,y mutations from each simulation’s s distribution at each adaptive step. The s distributions were generated during the mutation screen experiments. nx,y was min(ns>0x,y,100), where ns>0x,y was the number of mutant screen alleles with s > 0 in replicate x and adaptive step y. The sampled distributions were fit to a GPD as per (A), generating a distribution of κ from each replicate and adaptive step. The mean was again treated as the estimate for the shape parameter. (TIFF) [file pgen.1011289.s007.tiff]

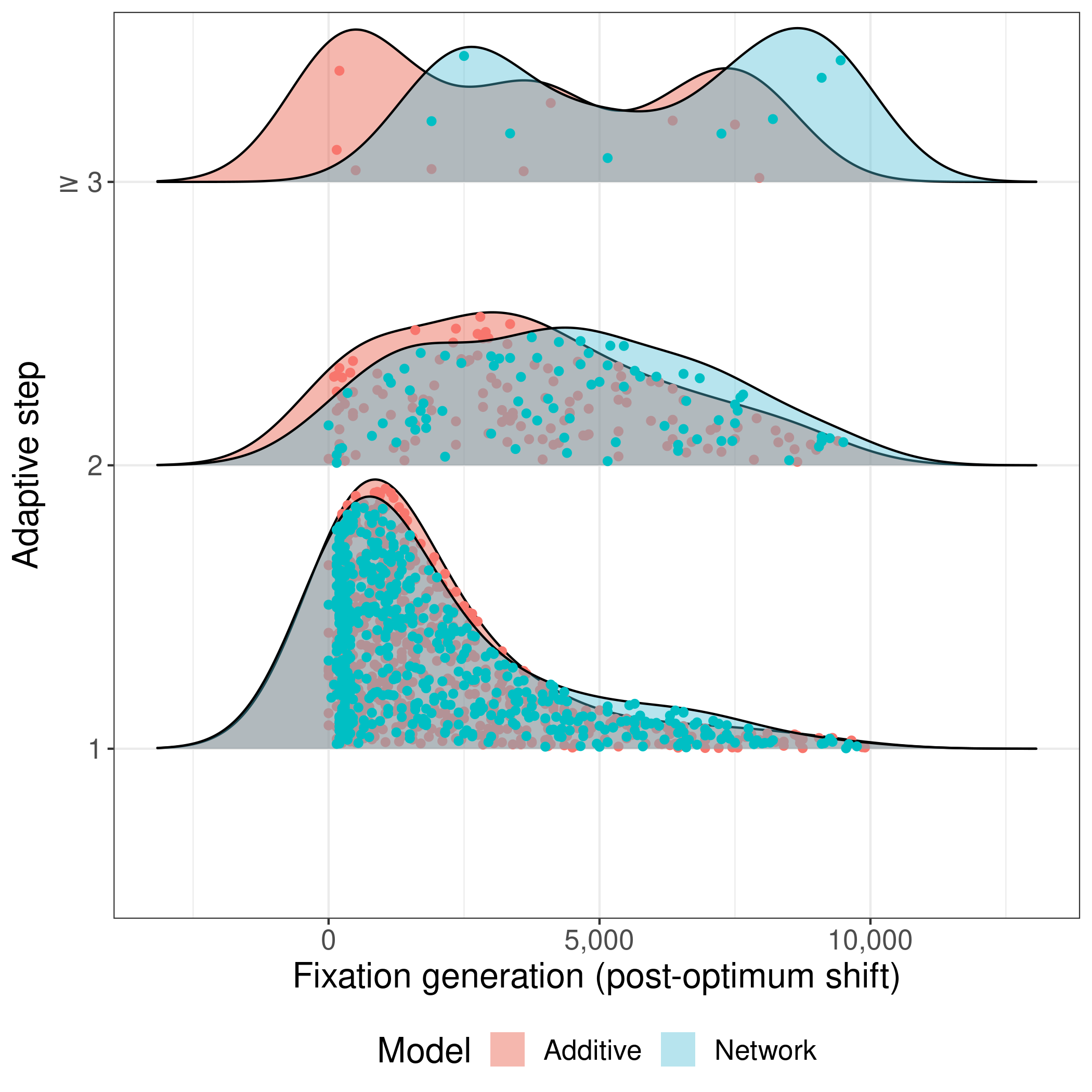

Supplement: S5 Fig — There was no significant difference between populations at adaptive step 1. Generations are given relative to the optimum shift so that Generation 0 is when the optimum shifted. (TIFF) [file pgen.1011289.s008.tiff]

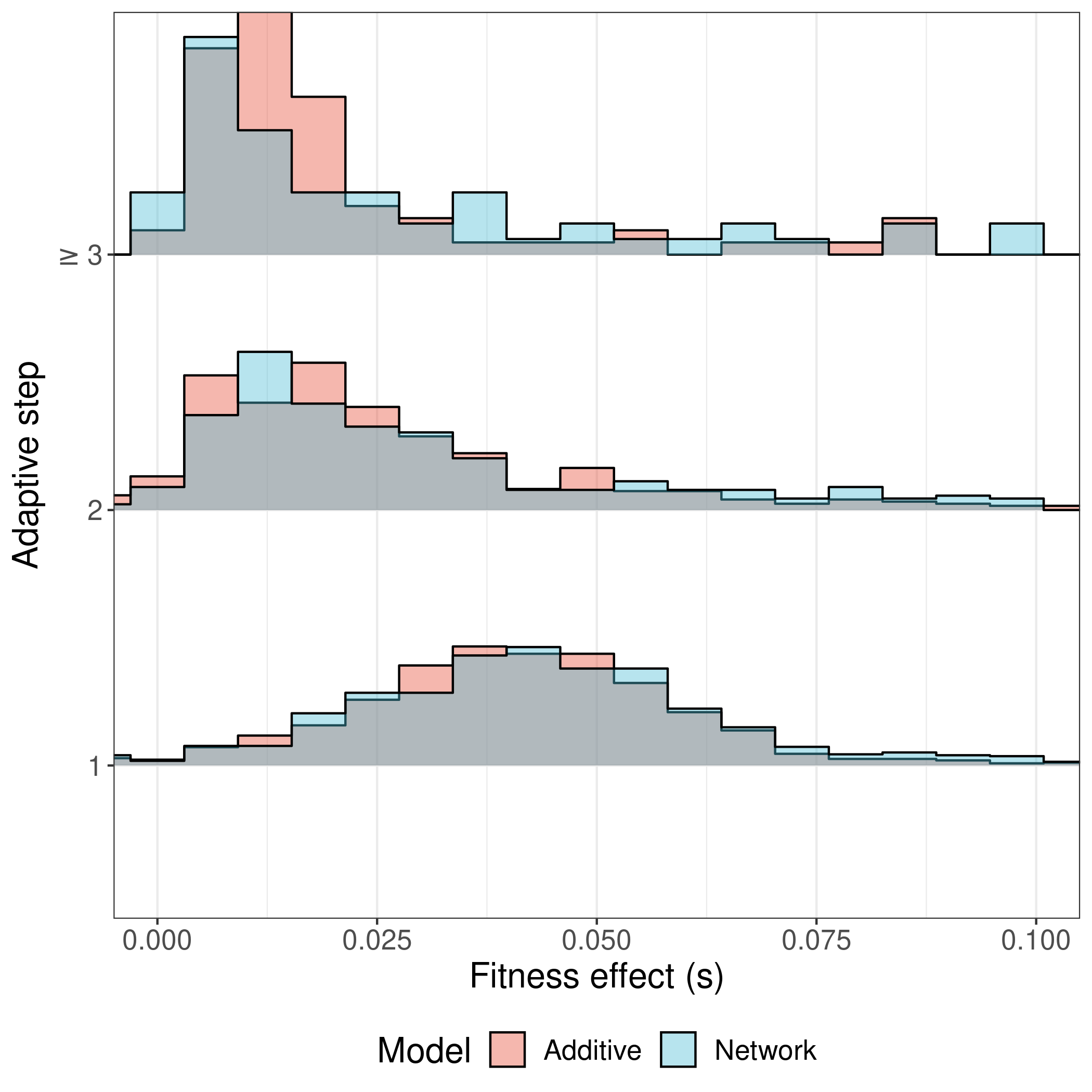

Supplement: S6 Fig — The mode of the distribution is clearly greater than 0, possibly due to small effect alleles being more susceptible to loss by drift. (TIFF) [file pgen.1011289.s009.tiff]

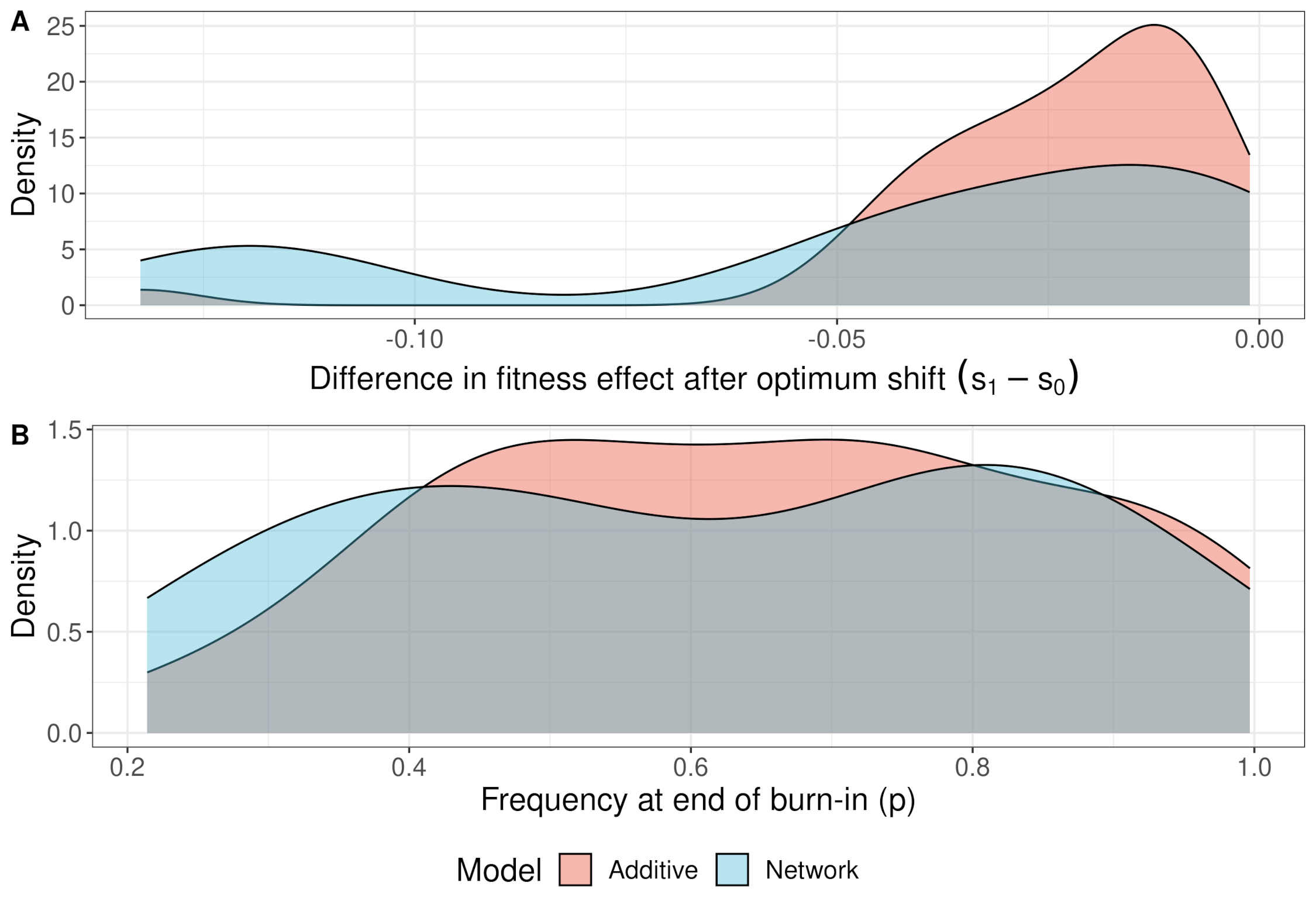

Supplement: S7 Fig — (A) The selection coefficient, s, decreases after the optimum shift at generation 50,000. (B) The allele frequency, p, of the fixations at the optimum shift. (TIFF) [file pgen.1011289.s010.tiff]

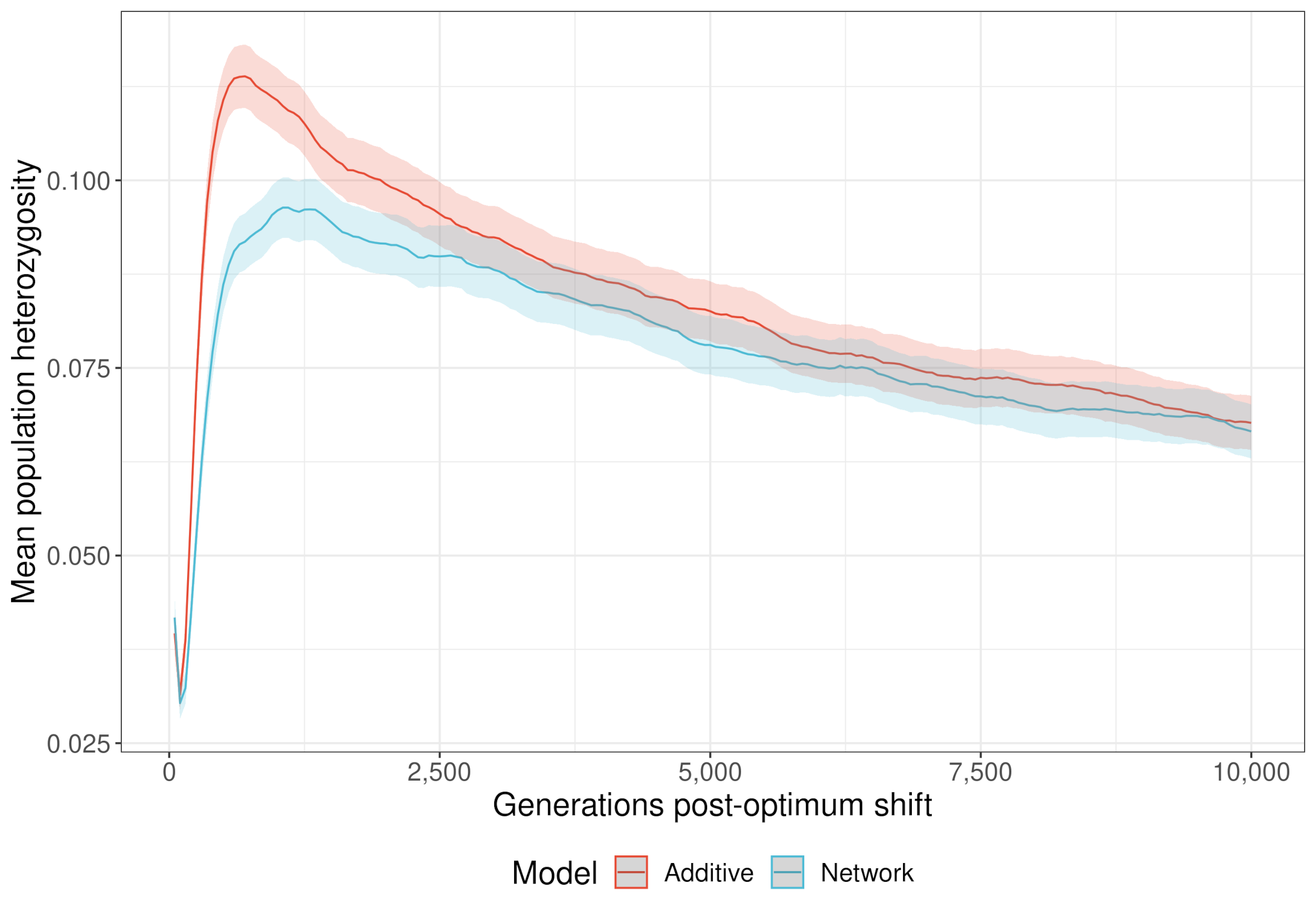

Supplement: S8 Fig — The mean is taken over 2,880 replicates per group. Heterozygosity was measured at the two QTLs (mQTLs in NAR models) in both models. (TIFF) [file pgen.1011289.s011.tiff]

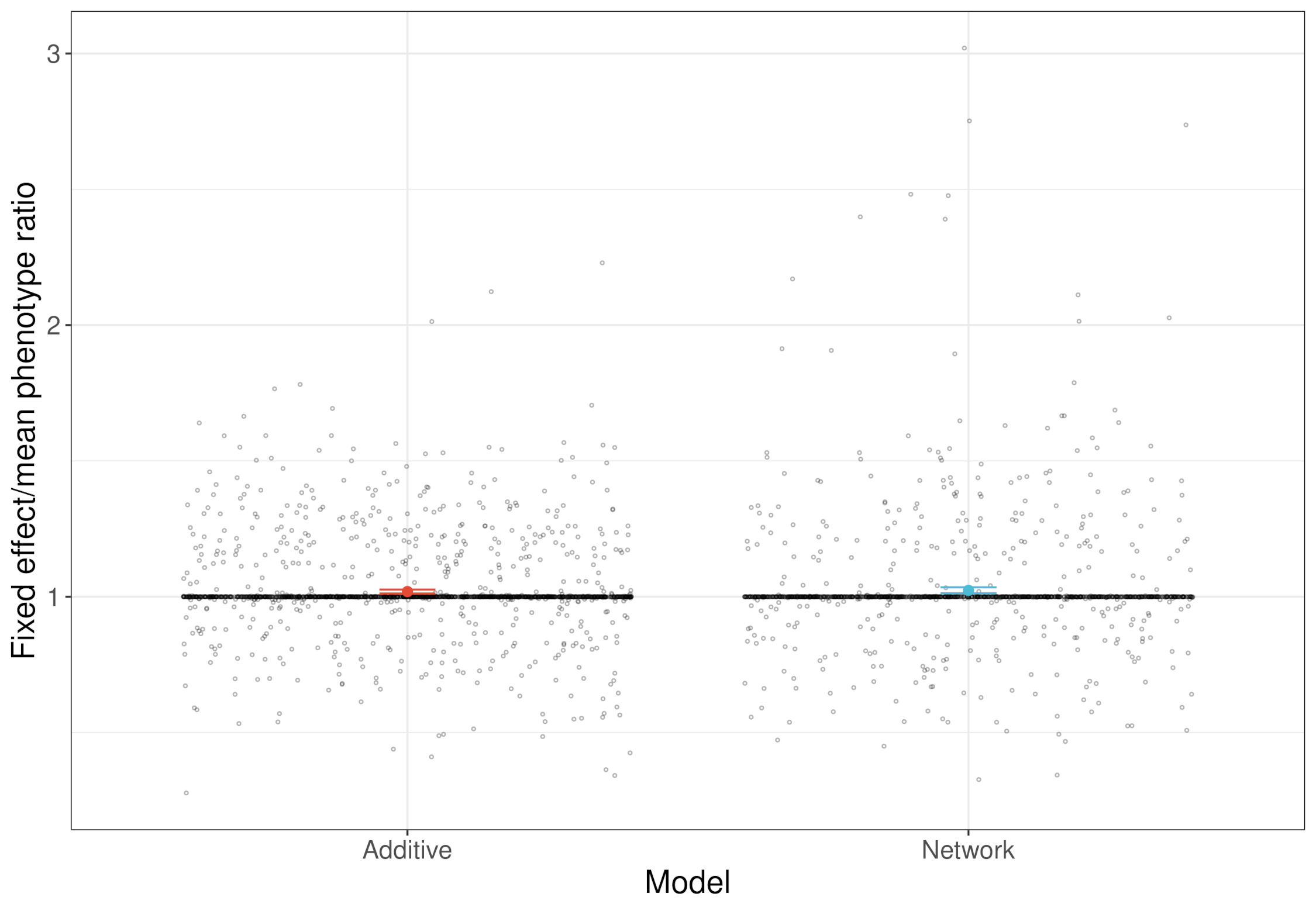

Supplement: S9 Fig — Values > 1 indicate that segregating variation decreases the mean population phenotype, and vice-versa for values < 1. Values = 1 indicate no segregating variation contributing to trait variance in the population. (TIFF) [file pgen.1011289.s012.tiff]

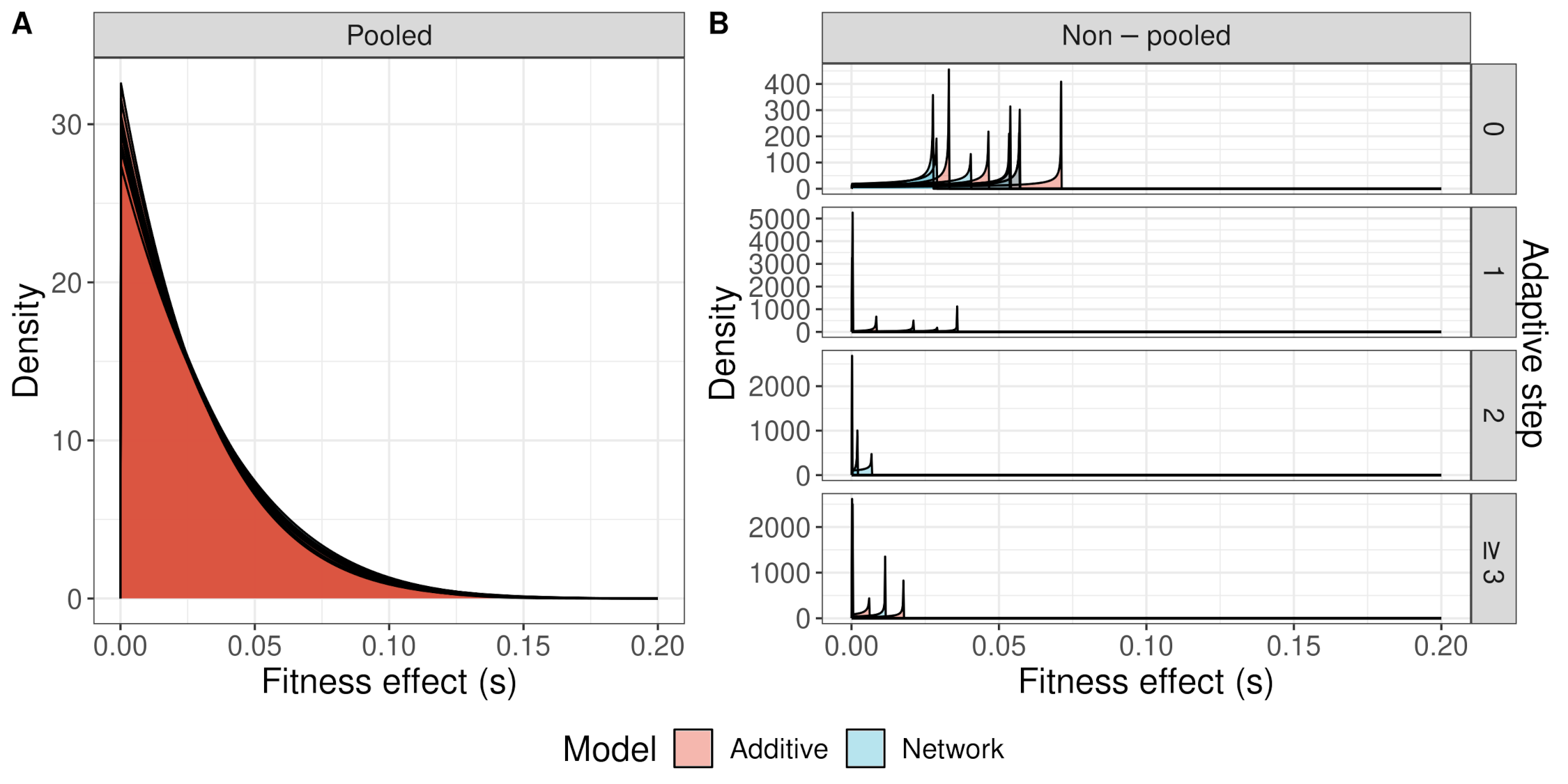

Supplement: S10 Fig — Mutations were created by a mutant screen experiment where 1,000 mutations were randomly generated and their fitness effects measured relative to the phenotype of a population at a given adaptive step. The pooled method (A) pooled together the mutations from all mutant screens across simulation replicates and adaptive steps and used bootstrapping to repeatedly sample 1,000 alleles from the pooled distribution to fit a GPD onto. The non-pooled method (B) fit the GPD onto a sample of 100 mutant screen alleles from each replicate simulation at each of its adaptive steps. Each figure shows the resulting GPD fits of 5 randomly sampled replicates per model of these two methods (i.e. either 5 simulations in the non-pooled case or 5 repeated samples of 1,000 alleles for the pooled case). Note that fewer than 5 samples existed for adaptive steps > 1 in (B), owing to the rarity of walks that long. (TIFF) [file pgen.1011289.s013.tiff]

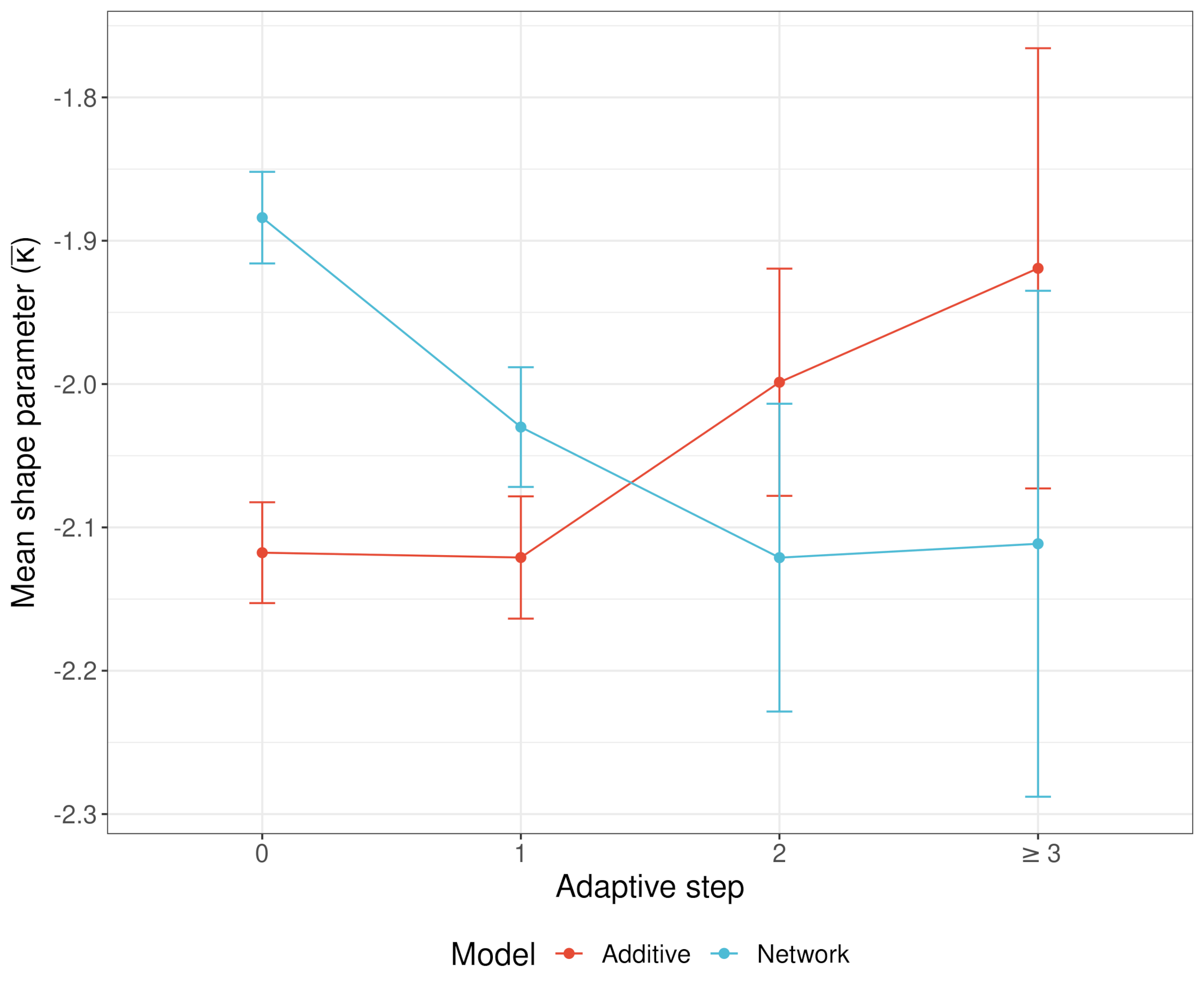

Supplement: S11 Fig — κ describes the shape of the GPD, with negative values (seen here) indicating a Weibull domain of attraction. As κ decreases, the maximum size of a beneficial mutation decreases. Error bars are 95% confidence intervals. Sample sizes for each group are given in S2 Table. (TIFF) [file pgen.1011289.s014.tiff]

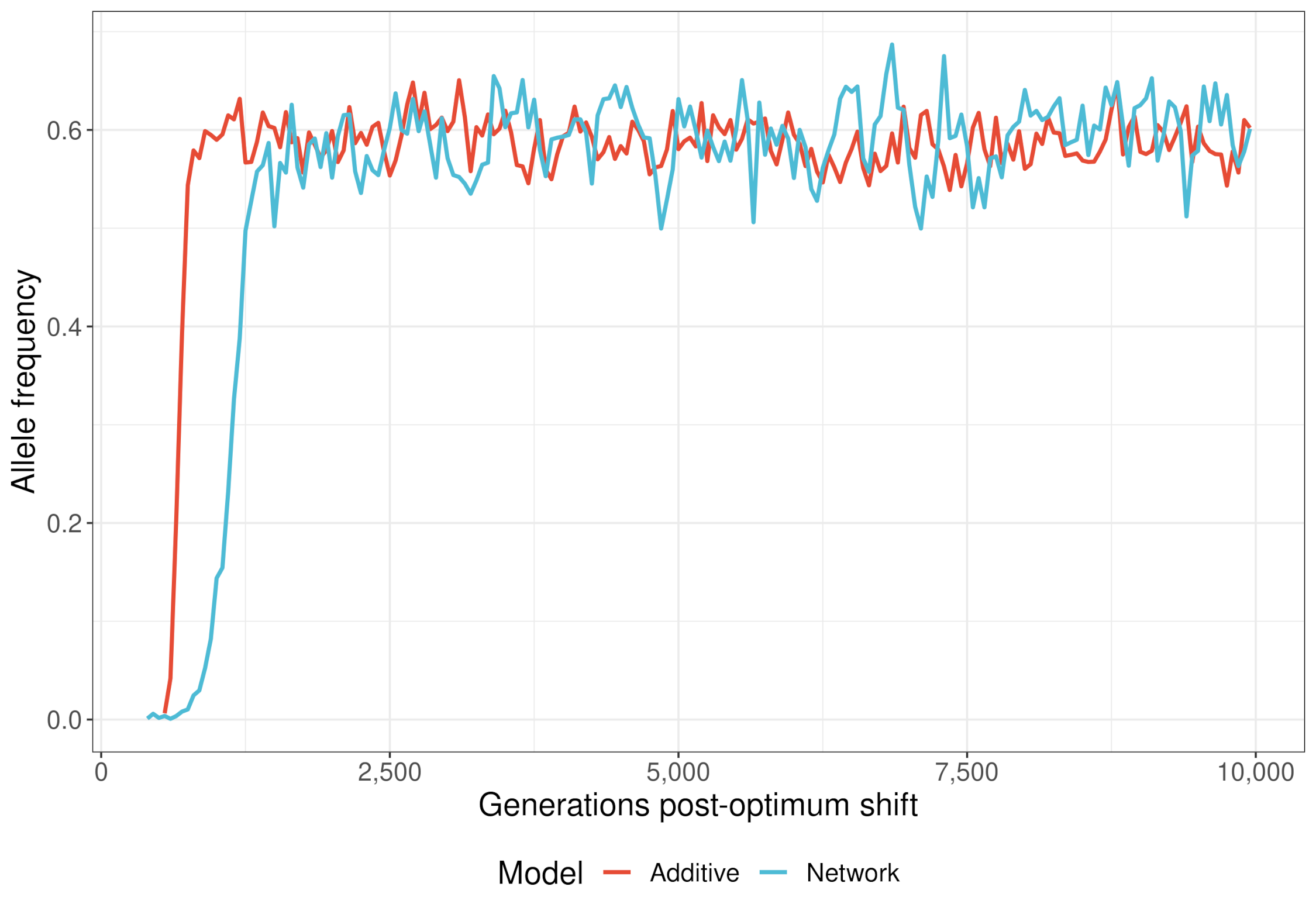

Supplement: S12 Fig — The additive mutation had a phenotypic effect α = −0.618 while the NAR allele had a phenotypic effect α = −0.689. (TIFF) [file pgen.1011289.s015.tiff]

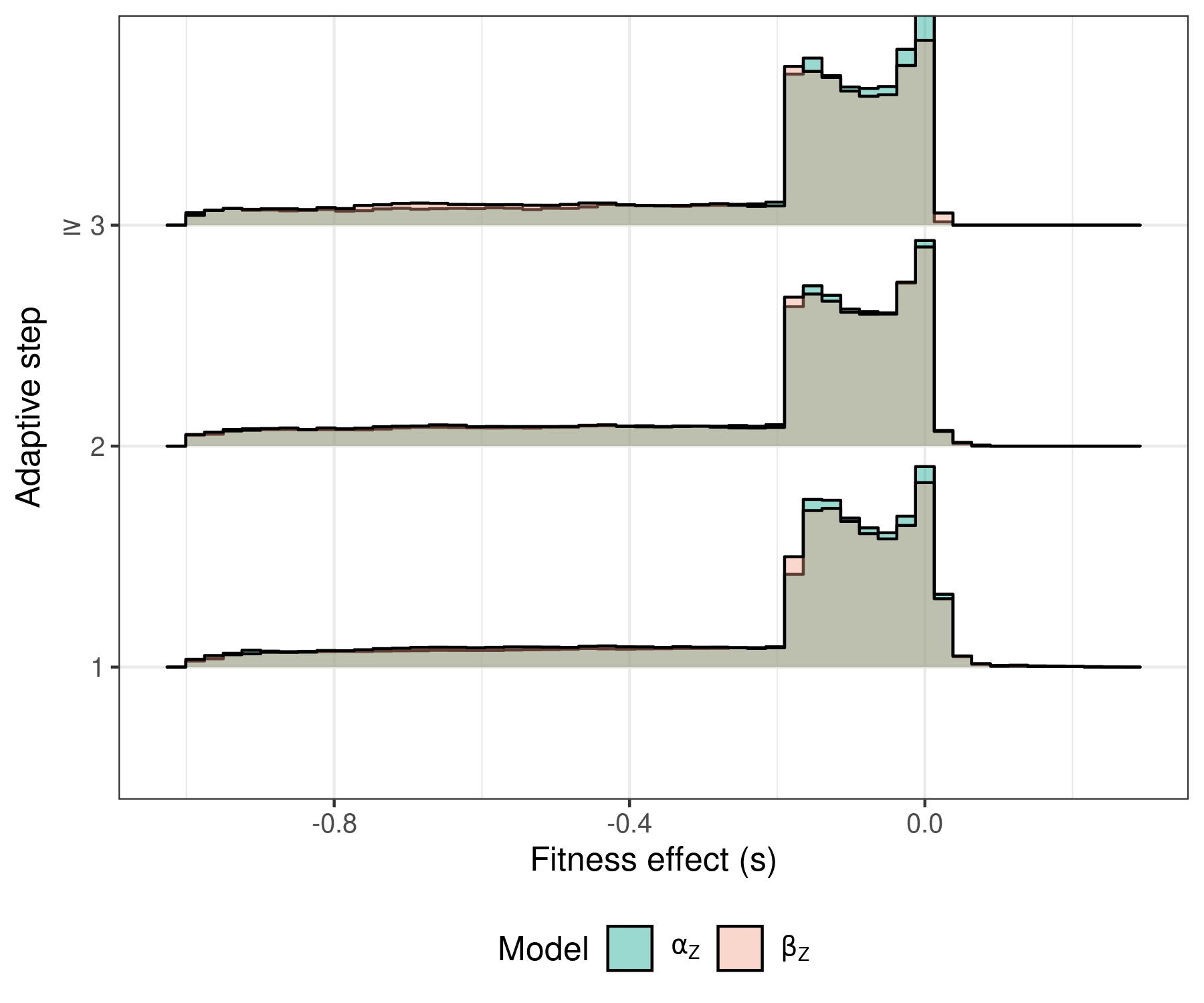

Supplement: S13 Fig — Compared to βZ mutations, αZ mutants were less likely to have strongly deleterious effects and more likely to have slightly deleterious effects existing on one of the two modes of the distribution. The shape of the distribution was similar between the molecular components. (TIFF) [file pgen.1011289.s016.tiff]
