# Supplementary material for: The distribution of fitness effects during adaptive walks using a simple genetic network
Source: PLoS Genet. 2024 May 24;20(5):e1011289. doi: 10.1371/journal.pgen.1011289 (PMC11156440; doi:10.1371/journal.pgen.1011289)
Supplement: Attachment — Submitted filename: PGENETICS-D-24-00130_ACCILIEN_REVIEW.docx [file pgen.1011289.s017.docx]

Fitness landscapes are a great tool to help understand how molecular evolution proceeds. This approach has been fruitful in yielding various results that allow us to explain the evolutionary dynamics that we observe in nature. However, the simplicity of these landscapes, while a useful feature, also presents certain limitations in how biologically realistic they can be. In my view, the strength of this paper is that it adds that layer of biological complexity by bringing together concepts from molecular biology (gene expression and regulation), quantitative/population biology (genotype-phenotype-fitness (or GPW) maps), and evolutionary genetics (selection, adaptive walks). While the paper itself only explores a limited subset of the parameter space that their model allows due to computational limitations, the general framework promises to yield further useful insights.

The authors found that additive and network models were both able to adapt within the prescribed number of generations under selection. However, more populations in the additive model were able to adapt relative to the network model. In addition, the network model took longer to adapt. Both took a similar number of adaptive steps which matched the prediction made by Orr 2003. The effect sizes of beneficial and fixed mutations were also similar across both models however, the distribution of fitness effects of new mutations differed strongly between the two models, with network populations being bimodal and skewing left due to excess deleterious mutations compared to additive populations. This excess of deleterious mutations might explain why fewer populations were able to adapt in the network model.

Overall, this paper has contributed to our knowledge of how we model adaptation. A key deficit in current fitness models is the way that complexity and gene interactions are modeled. Interaction effects, for example, are attributed to “noise” in an overall additive model. While this standard approach is seemingly adequate in a theoretical context, it becomes less so as we attempt to extrapolate those findings into biological systems. Explicitly modeling gene interactions, as the authors have done here, allows us to fine-tune more precisely specific types of interactions and explore their effects on the fitness landscape. That is why I think that the main contribution of this paper is the general framework that it provides for modeling genetic interactions. In addition, the results from the simple NAR network that it explores also offer some interesting insights. Namely, the fact that the frequency of adaptation was lower in the network than additive model. This suggests that the more common, oversimplified models are likely overestimating the probability of adaptation.

I did not identify any outstanding weaknesses. As previously stated, much work remains to be done with this model in exploring the effect of other molecular components other than $\alpha_{Z}$ and $\beta_{Z}$. Those can be addressed in subsequent publications.

A couple of minor points:

- A lot of time was spent discussing previous literature in the discussion. That, while useful information, might be more appropriate in the introduction or even in a supplementary discussion. I suggest tuning the discussion from the current literature review style to more of a discussion of how these results change our understanding of or approach to molecular evolution.
- There is no information on what the additive landscape is like. Figure 6 should include an additional panel of the additive landscape for comparison to the network landscape.
- Can landscape ruggedness be calculated? (e.g. departure from additivity, number of peaks, r/s ratio, correlation of fitness effects, etc.) While a qualitative description of the landscape is good, I would like to see a more quantitative approach taken as well. This would allow for comparison to other empirical and/or theoretical landscapes in the broader literature.
- Fig 2: What is the justification for $t_{start}$, $t_{stop}$, and $t_{max}$ in Eq. 2 and 6?
- Fig 3: Could probably do with fewer “Generations post optimum shift” panels. Would make the figure less crowded.
- Fig 5: Additive step “≥3” additive model bin off page
- Fig 3-5: I don’t understand why the overlap of the two models is necessary (grey bins). It obscures the differences.
- Fig 6C: Should y-axis be “Fitness” instead of “Phenotype”?
- Fig S11: Why is the 95% CI at step 2 and ≥3 so large in the network model?
