# Supplementary material for: The distribution of fitness effects during adaptive walks using a simple genetic network
Source: PLoS Genet. 2024 May 24;20(5):e1011289. doi: 10.1371/journal.pgen.1011289 (PMC11156440; doi:10.1371/journal.pgen.1011289)
Supplement: Attachment — Submitted filename: OBrien_response.docx [file pgen.1011289.s018.docx]

# *Response to reviewers*

*Here we respond directly to the reviewers’ comments. All line numbers referenced are for the marked-up manuscript (file OBrien_etal_2024_markup.pdf), not the clean revision (file OBrien_etal_2024_rev.pdf).*

## Editor’s comment

Three reviewers (and I) have read your manuscript entitled "The distribution of fitness effects during adaptive walks using a simple genetic network" and all reviews were largely positive. This detailed simulation study appears to be very carefully conducted and contains several interesting findings that will be of interest to geneticists studying adaptation and gene regulatory networks. Several reviewer points should be addressed in a minor revision. One that I think is particularly important is working on readability for a broad audience. The subject matter is difficult, and overall, authors have done an excellent job of explaining the context, approach and results. Two specific suggestions are to reduce acronyms (these are distracting and similar enough that it is difficult to keep them all straight), and to reduce subscripts that are unnecessary (see R1's comments). It would also be useful to present the full set of differential equations prior to equation 1. R1 also asks for a justification of using SLIM when the SSWM model and simple network might allow for a simpler (and more computationally efficient) approach. R2 makes important points about the relevance of the cost of complexity discussion when the network has only 2 genes and is in the SSWM parameter space. R2 also notes that the dependence of the distribution of fitness effects on evolutionary history has limited relevance to the question - and this could be mentioned. R3 has several suggestions about comparisons and calculations of fitness landscapes.

- *We thank the editor for their suggestions. In the revision we have made numerous clarifications to improve the broad appeal of the paper. We have addressed each of the reviewers’ comments. Regarding moving the differential equations from the methods to earlier in the manuscript, we did this in an early version of the paper but found that readers with a less mathematical background had a hard time following the section.*

## Reviewer #1

1.	O'Brien et al. use a simple gene network model to investigate the distribution of fitness effects (DFE) during the course of adaptive evolution. They model a quantitative trait under stabilizing selection determined by the negative autoregulation (NAR) network motif. They run evolutionary simulations to investigate adaptive walks following a shift to a new phenotypic optimum and use a simple additive model as a control. O'Brien et al. find that the NAR and additive models behave in a broadly similar manner but with some interesting differences. For example:

(a) Populations adapted more slowly under the NAR model than under the additive model.
(b) The DFEs of populations adapting under the NAR model had fewer beneficial mutations than those of populations adapting under the additive model.

The research is topical and is likely to appeal to a broad audience of geneticists (evolutionary, population, quantitative, systems, etc). The methods are sound. The paper is clearly written and well-argued.

- *We thank the reviewer for their* *positive comments.*

2.	In lines 41 and 46 you write that some distribution is "analogous" to another distribution? I don't understand what that means and to what end the analogies are being made.

- *The aim of this section was to make it clear that the distribution of fitnesses among genotypes is different to the distribution of fitness effects among mutations, but they are related. The beneficial genotypes (those to the right of the wildtype in the distribution of fitness values) contain beneficial mutations. We have revised the confusing phrases and added clarification (lines 38-53).*

3.	Since your study was conducted in the SSWM regime (line 334), was there a reason not to use the more efficient approach of simulating an adaptive walk by sampling a candidate mutation and accepting or rejecting it as a substitution based on the probability of fixation? If so, the reason should be stated.

- *This would be a much more efficient approach for this scenario. However, we developed our model with the goal of producing a robust framework to study many evolutionary scenarios and genetic architectures, including those that fall outside the SSWM regime. We outlined this general model in the supplementary material. We have added a comment on this at line 242-250.*

4.	There are a lot of subscripts. The subscript in H_O (Eq. 7) seems unnecessary since no other kind of heterozygosity is used. Also, it is confusing because the subscript O is used to designate "optimal" in P_O (line 271).

- *We agree that the O subscript is unnecessary and have removed it (line 366-367, and ‘Assumption validation’ section starting line 496).*

5.	Another subscript that may not be needed is "fs" in r_fs (Eq. 8). Also, the subscript is capitalized in the Results (e.g., line 462) but not in Eq. 8.

- *We have removed the ‘fs’ subscript from r_{fs} (line 375, ‘Assumption validation’ section starting line 496).*

6.	Are the effects of mutations described in lines 347-379 assayed in a heterozygous or homozygous state? Please clarify.

- *We assayed mutations in homozygous state – we’ve clarified this on lines 387, 390 and 413.*

7.	In line 786 you write: "There are other motifs that are common in nature that could be reasonable choices for a toy model of network-mediated traits. Other options include feedback loops, feedforward loops..." You appear to have missed a pioneering study of compensatory evolution using a model of the type I coherent feedforward loop network motif from a decade ago.

K. Bullaughey. Multidimensional adaptive evolution of a feed-forward network and the illusion of compensation. Evolution 67(1): 49-65, 2013.

I think you'll find it interesting.

- *We thank the reviewer for bringing this paper to our attention. It is interesting indeed! We have included it as a citation as an example of feed-forward loop architectures in evolution (line 870-872).*

8.	In line 830 you mention that some alleles were observed to experience balancing selection. Presumably these were cases of overdominance. I know this is beyond the scope of this paper but I would be interested to see the distribution of dominance effects (see also my comment #4).

- *We agree that the distribution of dominance effects would be an interesting case to explore in a future study, perhaps more focused on polygenic interactions where these effects can have a larger effect on adaptation (and where effects are more likely to be heterozygous). It is unfortunately out of scope for this paper, as you identified.*

## Reviewer #2

1.	This paper appears to be a very carefully developed and produced computational study. I have only minor comments on what the paper presents. But there are issues with what the paper doesn't present that I will comment on. I will not be asking that any of the work in the submission be re-done, but only that the issues I raise be addressed in the manuscript.

- *We thank the reviewer for their helpful comments.*

2.	The introduction to Gillespie-Orr theory is welcome. Many papers on the subject have been published. So the following issue is not unique to this submission. The assumption behind Gillespie-Orr theory is that one is taking i.i.d. samples from some probability distribution. But there is no mechanistic basis why that model should be appropriate for the mutational neighborhoods of organismal genotypes.

- *This is an important point that we have now included in the discussion (line 915-923). We chose the Gillespie-Orr framework because the vast majority of DFE studies have employed/assumed this model and this enables more obvious parallels to be drawn between this study and previous work.*

3.	There is an important point never mentioned in the paper, which is that the dependence of the DFE on the evolutionary history enters ONLY as it sets the values of \alpha_Z and \beta_Z. In other words, \alpha_Z and \beta_Z screen-off (Salmon 1971) the prior history of the population. So all we need to find out about the DFE is just to calculate it over the the different points on the 2-D fitness landscape. To sample it only for the endpoints of evolutionary trajectories gives us only some snapshots of the DFE. So question of whether or not the SSWM regime persists is irrelevant to the DFE except in how it affects the evolutionary trajectories of \alpha_Z and \beta_Z.

- *This is true, but note that although the ‘full’ DFE contains all the possible mutations that a population could use, it does not reveal which mutations are readily available to the population at a given time during an adaptive walk. For instance, when a population is far from the optimum, there is a (small) proportion of beneficial mutations. However, a population perfectly positioned at the optimum only has deleterious mutations available. Hence, the sampling of the DFE at different timepoints of the walk give a representation of the DFE of mutations available to the population at that time, which is much more important for an adaptive walk than the total DFE across the entire fitness landscape (which includes inaccessible molecular component combinations). The mutational effect variance parameter (table 1, pg 10) controls the size of the subspace of available mutations (or the size of the region of the fitness landscape explorable within a single step). We have included this point in the methods along with the fitness landscape estimation methods (lines 456-465).*

4.	Mutation consists of i.i.d. perturbations of the \alpha_Z and \beta_Z values, which the dynamics map to fitnesses. Because the fitnesses are bounded above, if any extreme value distributions are applicable it would be the Weibull Type III.

- *This is a feature of stabilizing selection and is why we see the shape parameter decrease over the walk. However, early in the walk, when populations are sufficiently far from the optimum, they face directional selection so that the EVD might approximate a different shape. However, the extent to which this shape might differ depends on the initial distance to the optimum, which we have mentioned on line 710-712.*

5.	With only two parameters evolving, it is hard to see how the results here have any bearing on the "cost of complexity" issue brought up many times throughout the paper.

- *While it is true that the NAR system is very simple, we were surprised by the complexity that emerged in the NAR DFE relative to the additive simulations. This suggests that the cost of complexity might be of more importance than previously thought. We have clarified this idea in the discussion (line 791-796).*

6.	p. 20 discusses the evolution of more complex gene networks. There is a substantial literature on the evolvability of gene networks that appears divorced from the current study. Indeed "evolvability" appears nowhere in the paper.

- *We agree that evolvability in genetic networks is a large body of work, although it is typically focused on the addition and removal of nodes in the network (via gene duplication and subsequent neo/subfunctionalisation). Our model is more focused on mutations which change edge weights in the network and so is different to the evolvability literature in this regard. This point is implied in the introduction (in comparison to Paul Francois’s work – see reference list), however we have now made a direct mention of evolvability to make this connection clearer (lines 178-179).*

Minor Comments:

7.	The paper never explicitly states that L_Q = 2 in all the simulations. While we learn that it is 2 genes on p. 4 line 127, nowhere is L_Q = 2 stated. Supplement Fig 1 is misleading in that it shows more than 2 genes but makes no mention that in the simulations, only 2 are considered.

- *We thank the reviewer for pointing out this oversight. We have now stated L_Q = 2 in the methods (lines 272-273). S1 Fig 1 shows the general hierarchical model for any number of loci. We mention this when we refer the reader to the general model (lines 242-250).*

8.	p. 6 line 189: It is hard to parse K_{XZ}^{n_{XZ}}, etc. If you can find a way to make it more readable, that would be good. Also, K is never defined.

- *We have defined K and renamed/combined n_XZ and n_Z to h, since they have the same value in our study (lines 202-208). We have also added a sentence to the discussion to mention that in future h can be split into separate parameters for X and Z (line 840-843).*

9.	p. 6 line 213: "The constant values of the other..." That should be moved up to the first appearance of the symbols to indicate they are not varied.

- *To maintain the flow of the section, we have split the ODE description into two paragraphs: one for the fixed parameters, and one for \alpha_Z and \beta_Z. We moved and reworded the mentioned sentence to line 208 to make clear that the fixed parameters are constant.*

10.	p. 7 line 247. The first appearance of L_Q. Here is where it should be stated that all the simulations run in the paper assume L_Q = 2. If that is not correct, then I am really stymied as to what values L_Q takes in the work.

- *We have done this as per the first of Reviewer 2’s minor comments (line 272-273).*

11.	p. 8 line 283: "per locus per generation" The reader should be reminded that there are only 2 loci in the simulations.

- *We have added this reminder at line 311-312.*

12.	p. 8 line 310: "the optimum shift". This is the first we are told of this. It is so fundamental that it shouldn't be buried in the middle of a middle paragraph. There is a fundamental difference between populations near mutation-selection balance and those that are far from balance, so the presence of an optimum shift, and its magnitude, are core aspects of the model. It should be included in the Intro on p. 2.

- *We agree and have added a mention of optimum shifts in our first paragraph (line 23-27) and at the end of the introduction (line 186).*

13.	p. 9 Table 1. It should include L_Q.

- *We have added the missing parameter to Table 1 (p*g *10).*

14.	p. 9 The section describing H_O^i and r_{fs} never explain how they will be used to validate the SSWM conditions. Something should be stated here.

- *We added some information on what these values should be under SSWM in both the methods section and the results (lines 371-372, 378-380, 512-513)*

15.	p. 10 line 348: "An important part of adaptive walk theory is the shape of the DFE." This is the whole purpose of the paper so it is a bit odd to call it "an important part".

- *Good point, we have reworded the sentence (line 382).*

16.	p. 10 line 357: eq (9). In adaptation on a fitness landscape, what enters the dynamics is not the absolute difference in fitnesses, but the relative increase. So I would think a better definition is s = (w_k / w) - 1.

- *We investigated this suggestion and found that the relative approach was similar to the absolute difference (s = w – w_k) we had originally calculated. This was likely due to the position of the optimum: s_rel = (w / w_k) – 1 = (w – w_k)/w_k, so that when w_k ~ 1, s_rel ~ s_abs. Note that we consider the reciprocal of your definition (w/w_k instead of w_k/w) for correct signage (fitness of an individual with a mutation, w, relative to fitness of an individual without that mutation, w_k). Because w_k was ambiguous, we have also reworded the fitness effect calculation description with w_aa and w_AA representing w_k and w, respectively (lines 385-393). For the sake of reducing complexity, we think that the absolute measure is simpler to understand and thus we stay with this measure. Below is a figure showing a regression of the two:*

*We believe the equivalency between the two measures arises because fitness is relative in this study, such that s_rel = (w – w_k) / w_k produces very similar outcomes to (w - w_k), especially when w ~ 1 (which it usually is, owing to the position of the optimum). We have reiterated that we use relative fitness in the methods (lines 357, 386-391, Table 1 pg 10).*

17.	Fig. S10B: The curves all look like hypberbolas. Is that real or artifact or what? They are so sparse that I would not want to infer that they belong to any particular distribution type.

- *That is a product of the estimated shape parameters for the GPD: when kappa < -1, there is a limit on the size of positive effects. The hyperbolic ‘spike’ is an artifact of this limit.*

18.	p. 21 line 847. "The strong selection and weak mutation assumptions grant computational tractability"
Since the paper uses individual-based simulations with the Wright-Fisher model, the most computationally intensive aspect is already included. There would be no added computational cost to shifting to strong mutation.

- *This is not entirely true, since increasing mutation rates increases memory costs and computational requirements for storing and searching arrays of mutation objects in SLiM. From our unpublished experiments, high mutation rates do affect computation time, however you are correct that this isn’t a showstopping performance impact. We have reworded the sentence to be a clearer that SSWM improves computational efficiency rather than making it tractable at all (lines 928-929).*

19.	Supplemental figures: The figure labels should be on the graphic itself, not just the separate file titles. It is a bit annoying for reviewers to have to piece together the paper from the main file, supplement file, and supplemental figure files.

- *We apologize to the reviewer for this and understand the inconvenience this caused: we will embed captions with the supplementary figures for reviewers in the future.*

20.	p. 22: The points about future computational savings are well taken. The authors already computed the adaptive landscape of the two variables \alpha_Z and \beta_Z. It should be possible to closely approximate that with smooth functions, or even a deep neural network, to emulate the ODEs, and save on computation.

- *We thank the reviewer for their astute comment and have included these recommendations for future improvements at lines 955-956.*

## Reviewer #3

1.	Fitness landscapes are a great tool to help understand how molecular evolution proceeds. This approach has been fruitful in yielding various results that allow us to explain the evolutionary dynamics that we observe in nature. However, the simplicity of these landscapes, while a useful feature, also presents certain limitations in how biologically realistic they can be. In my view, the strength of this paper is that it adds that layer of biological complexity by bringing together concepts from molecular biology (gene expression and regulation), quantitative/population biology (genotype-phenotype-fitness (or GPW) maps), and evolutionary genetics (selection, adaptive walks). While the paper itself only explores a limited subset of the parameter space that their model allows due to computational limitations, the general framework promises to yield further useful insights.

The authors found that additive and network models were both able to adapt within the prescribed number of generations under selection. However, more populations in the additive model were able to adapt relative to the network model. In addition, the network model took longer to adapt. Both took a similar number of adaptive steps which matched the prediction made by Orr 2003. The effect sizes of beneficial and fixed mutations were also similar across both models however, the distribution of fitness effects of new mutations differed strongly between the two models, with network populations being bimodal and skewing left due to excess deleterious mutations compared to additive populations. This excess of deleterious mutations might explain why fewer populations were able to adapt in the network model.

Overall, this paper has contributed to our knowledge of how we model adaptation. A key deficit in current fitness models is the way that complexity and gene interactions are modeled. Interaction effects, for example, are attributed to “noise” in an overall additive model. While this standard approach is seemingly adequate in a theoretical context, it becomes less so as we attempt to extrapolate those findings into biological systems. Explicitly modeling gene interactions, as the authors have done here, allows us to fine-tune more precisely specific types of interactions and explore their effects on the fitness landscape. That is why I think that the main contribution of this paper is the general framework that it provides for modeling genetic interactions. In addition, the results from the simple NAR network that it explores also offer some interesting insights. Namely, the fact that the frequency of adaptation was lower in the network than additive model. This suggests that the more common, oversimplified models are likely overestimating the probability of adaptation.

I did not identify any outstanding weaknesses. As previously stated, much work remains to be done with this model in exploring the effect of other molecular components other than $\alpha_{Z}$ and $\beta_{Z}$. Those can be addressed in subsequent publications.

- *We thank the reviewer for their positive comments and are pleased that the main arguments of the manuscript were able to come through clearly for them.*

2.	A lot of time was spent discussing previous literature in the discussion. That, while useful information, might be more appropriate in the introduction or even in a supplementary discussion. I suggest tuning the discussion from the current literature review style to more of a discussion of how these results change our understanding of or approach to molecular evolution.

- *Since the paper integrates quantitative genetics, population genetics, and systems biology, we felt it necessary to link our results more closely to previous literature and show that our approach brings these fields together. We discuss how our model might change our understanding in the subsection “Opportunities and challenges in modelling complex genetic networks”.*

3.	There is no information on what the additive landscape is like. Figure 6 should include an additional panel of the additive landscape for comparison to the network landscape.

- *Since the additive landscape is 1D, it is just the fitness function, a normal distribution. We have added this point to the discussion on line 750-752.*

4.	Can landscape ruggedness be calculated? (e.g. departure from additivity, number of peaks, r/s ratio, correlation of fitness effects, etc.) While a qualitative description of the landscape is good, I would like to see a more quantitative approach taken as well. This would allow for comparison to other empirical and/or theoretical landscapes in the broader literature.

- *While we agree that a quantitative metric for ruggedness would be valuable in future studies using our method, we do not believe that it is necessary for this specific application given the simplicity of the network. Fig. 6A and B show that the fitness landscape of the two molecular components is ridgelike (i.e. fairly smooth – still more rugged than the additive, but simple regardless). In future studies we plan to investigate a wider range of molecular components in the NAR (e.g. the K parameters), and alternative networks with more complex interactions. In these cases, we believe that it would make more sense to quantify landscape ruggedness.*

5.	Fig 2: What is the justification for $t_{start}$, $t_{stop}$, and $t_{max}$ in Eq. 2 and 6?

- *We have added more detail for these parameters in the methods (lines 289-293). t_{max} was chosen to cover a reasonable range of phenotype solutions while avoiding unnecessary computation. t_{start} and t_{stop} were chosen so that X was activated half the total computed time.*

6.	Fig 3: Could probably do with fewer “Generations post optimum shift” panels. Would make the figure less crowded.

- *We agree: we have reduced the number of bins from 11 to 7.*

7.	Fig 5: Additive step “≥3” additive model bin off page

- *Thank you for pointing this out – we have adjusted the figure boundaries to fix this.*

8.	Fig 3-5: I don’t understand why the overlap of the two models is necessary (grey bins). It obscures the differences.

- *The idea of the grey of overlap is to make the differences between models more obvious: the coloured deviations from the grey show where the models differ from each other.*

9.	Fig 6C: Should y-axis be “Fitness” instead of “Phenotype”?

- *The idea of Fig 6C is to show the curvature of the genotype-phenotype-fitness map, the y axis is meant to be the phenotype, the x axis represents the genotype. We have fixed a typo in the figure caption that may have led to this confusion: “Fitness is a nonlinear function…” should have read “The phenotype is a nonlinear function…” .*

10.	Fig S11: Why is the 95% CI at step 2 and ≥3 so large in the network model?

- *The kappa estimation method we used (Beisel et al. 2007) can produce incorrect large magnitude kappa estimates when there are not enough mutations in the sample to properly fit a GPD. Beisel et al. recommend at least 10 mutations when kappa < 0, which we chose. Upon investigating this further, we found that 10 mutations did not produce consistent CIs for our dataset. Both additive and NAR populations had outliers with very large kappa estimates. The n >= 10 filter worked for estimates in additive models, but there were some invalid NAR estimates in the range of 10 < n < 20. Since there were relatively few populations that took more than 1 step to reach the optimum, the CI was strongly affected by these outliers. The means were only marginally affected. We have recalculated the averages/CIs, filtering out any estimates based on fewer than 20 mutations. We have updated Fig S11, the methods (line 488), results (line 599, Table S2, and the discussion (line 702 - 705) to reflect these changes. The conclusions from the kappa estimates have not changed, only minor changes to the estimates themselves and the aforementioned text, table, and figure. We thank the reviewer for highlighting this discrepancy to us.*

# *Additional changes*

*We list here additional changes that reviewers did not explicitly request. Line numbers are from the marked-up manuscript (file OBrien_etal_2024_markup.pdf). Changes not listed below nor in the response to reviewers were minor typographical or grammar changes made to improve the clarity of the document.*

- *Line 45-53: Replaced DBFE with DFE among beneficial mutations. This should remove confusion between the two similar terms. This change reoccurs throughout the manuscript.*
- *Lines 84-90: Removed acronyms that aren’t used repeatedly in the manuscript (PW, GW).*
- *Line 138-148: The paragraph was quite technical in its examples and language. We have removed some of this from the WRINKLED1 example and given context to the age of the NAR conservation for readers without a plant evolution background.*
- *Line 153-154: Combined similar sentences.*
- *Line 200: Added parentheses around Eq 1 Hill functions to reduce the chance the two fractions are misinterpreted as a single one.*
- *Line 298-299, Table 1: Removed unnecessary subscript from sigma*
- *Table 1 (pg 10): Removed extra caption, typeset long subscripts to use Roman text instead of italics.*
- *Line 427-429: Replaced n^{ISMS} with m and n_{s > 0}^{ISMS} with m_{s > 0} to reduce confusing acronyms.*
- *Lines 742-743 and 1265-1267: We have added a reference to a recent empirical paper on the distribution of fitness effects in Escherichia coli (Couce et al. 2024;* [*https://doi.org/10.1126/science.add1417*](https://doi.org/10.1126/science.add1417)*). Couce et al. found a similarly shaped DFE as the NAR in our paper, which we thought was interesting and timely to include.*
- *References: Improved consistency of journal abbreviations, added references to support additional information on adaptive walks (Cited: line 27, Ref: line 1011-1012), time of monocot-dicot divergence (see second bullet point; Cited: line 144, Ref: line 1153-1155), and the effects of correlations between mutations on Gillespie-Orr model (Cited: lines 916-917 and 921, Ref: line 1336-1343).*
